# Exogenous Activation of Invariant Natural Killer T Cells by α-Galactosylceramide Reduces Pneumococcal Outgrowth and Dissemination Postinfluenza

**DOI:** 10.1128/mBio.01440-16

**Published:** 2016-11-01

**Authors:** Adeline Barthelemy, Stoyan Ivanov, Maya Hassane, Josette Fontaine, Béatrice Heurtault, Benoit Frisch, Christelle Faveeuw, Christophe Paget, François Trottein

**Affiliations:** a CIIL—Centre d’Infection et d’Immunité de Lille, University of Lille, Lille, France; b Centre National de la Recherche Scientifique, UMR 8204, Lille, France; c Institut National de la Santé et de la Recherche Médicale, U1019, Lille, France; d Centre Hospitalier Universitaire de Lille, Lille, France; e Institut Pasteur de Lille, Lille, France; f Laboratoire Microbiologie Santé et Environnement, Université Libanaise, Tripoli, Lebanon; g Laboratoire de Conception et Application de Molécules Bioactives, Centre National de la Recherche Scientifique, Université de Strasbourg, Faculté de Pharmacie, Illkirch, France

## Abstract

Influenza A virus infection can predispose to potentially devastating secondary bacterial infections. Invariant natural killer T (iNKT) cells are unconventional, lipid-reactive T lymphocytes that exert potent immunostimulatory functions. Using a mouse model of postinfluenza invasive secondary pneumococcal infection, we sought to establish whether α-galactosylceramide (α-GalCer [a potent iNKT cell agonist that is currently in clinical development]) could limit bacterial superinfection. Our results highlighted the presence of a critical time window during which α-GalCer treatment can trigger iNKT cell activation and influence resistance to postinfluenza secondary pneumococcal infection. Intranasal treatment with α-GalCer during the acute phase (on day 7) of influenza virus H3N2 and H1N1 infection failed to activate (gamma interferon [IFN-γ] and interleukin-17A [IL-17A]) iNKT cells; this effect was associated with a strongly reduced number of conventional CD103^+^ dendritic cells in the respiratory tract. In contrast, α-GalCer treatment during the early phase (on day 4) or during the resolution phase (day 14) of influenza was associated with lower pneumococcal outgrowth and dissemination. Less intense viral-bacterial pneumonia and a lower morbidity rate were observed in superinfected mice treated with both α-GalCer (day 14) and the corticosteroid dexamethasone. Our results open the way to alternative (nonantiviral/nonantibiotic) iNKT-cell-based approaches for limiting postinfluenza secondary bacterial infections.

## IMPORTANCE

Despite the application of vaccination programs and antiviral drugs, influenza A virus (IAV) infection is responsible for widespread morbidity and mortality (500,000 deaths/year). Influenza infections can also result in sporadic pandemics that can be devastating: the 1918 pandemic led to the death of 50 million people. Severe bacterial infections are commonly associated with influenza and are significant contributors to the excess morbidity and mortality of influenza. Today’s treatments of secondary bacterial (pneumococcal) infections are still not effective enough, and antibiotic resistance is a major issue. Hence, there is an urgent need for novel therapies. In the present study, we set out to evaluate the efficacy of α-galactosylceramide (α-GalCer)—a potent agonist of invariant NKT cells that is currently in clinical development—in a mouse model of postinfluenza, highly invasive pneumococcal pneumonia. Our data indicate that treatment with α-GalCer reduces susceptibility to superinfections and, when combined with the corticosteroid dexamethasone, reduces viral-bacterial pneumonia.

## INTRODUCTION

Influenza A virus (IAV) infection can lead to secondary bacterial infections that can account for significant morbidity and mortality during epidemics and pandemics (1, 2). For instance, invasive bacterial pneumonia accounted for the majority of deaths (~40 million) during the 1918 Spanish flu pandemic (3, 4). One of the predominant causes of bacterial superinfections (e.g., during the 1918 Spanish flu and recent 2009 influenza pandemics) is *Streptococcus pneumoniae*, a group of extracellular encapsulated bacteria that often colonize the upper respiratory tract (5–7). In view of the threat of a highly pathogenic influenza pandemic and the emergence of bacterial antibiotic resistance, it is essential to gain a better understanding of the causes of (and treatments for) postinfluenza bacterial superinfections (8, 9). Enhanced vulnerability to secondary bacterial infections occurs between 4 days and several weeks after the primary influenza infection, depending on the pathogenicity of the virus and the status of the host’s immune system (3, 4, 10–12). Experiments with mouse models suggest that impairments in innate immunity contribute strongly to bacterial colonization and dissemination outside the lungs. In particular, loss and/or dysfunction of macrophages and neutrophils (key players in the clearance of extracellular bacteria) enhance susceptibility to secondary bacterial infections (13–17). Furthermore, disruption of the pulmonary (epithelial) barrier, exposure of new attachment sites, and impaired mucociliary function also contribute to postinfluenza bacterial superinfection (4, 9, 18–20).

Invariant natural killer T (iNKT) cells constitute a highly conserved subset of innate-like T lymphocytes with potent immunostimulatory properties. These cells recognize lipid antigens presented by the monomorphic major histocompatibilty complex (MHC) class I-like homologue CD1d expressed by antigen-presenting cells (21–24). Among the latter, conventional dendritic cells (DCs) are particularly well equipped to efficiently activate iNKT cells (25, 26). In response to T cell receptor triggering, iNKT cells rapidly release large amounts of Th1, Th2, and/or Th17 cytokines, which transactivate cells from the innate and adaptive immune systems (21–24). In view of this unique property, iNKT cells are critical for the regulation of innate and adaptive immune responses.

α-Galactosylceramide (α-GalCer) is a glycosphingolipid originally isolated from a marine sponge during a screen for antimetastatic agents (27). This molecule holds great promise as a cancer drug, and ongoing clinical trials are under way with the goal of optimizing antitumor properties mediated by iNKT cells (28–30). Prophylactic treatment with α-GalCer can also protect against many infectious diseases—including pneumococcal infections (21–24, 31–33). In the latter setting, the production of gamma interferon (IFN-γ) and interleukin-17A (IL-17A) by iNKT cells has a crucial role in bacterial clearance (32, 33). In the present study, we set out to evaluate the efficacy of α-GalCer and to determine whether it could minimize the harmful effect of combined IAV and *S. pneumoniae* exposure.

## RESULTS

### Influenza A virus infection induces a long-lasting susceptibility to secondary invasive pneumococcal infection.

**FIG 1:**
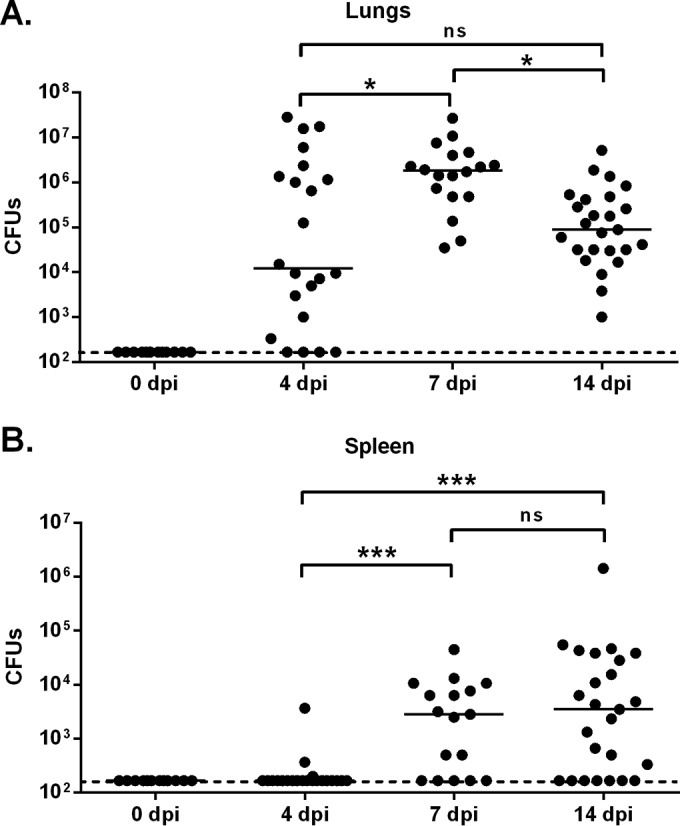
The dynamics of susceptibility to postinfluenza pneumococcal infection. Age-matched male mice were i.n. infected with IAV (30 PFU) or were left uninfected (0 dpi). Four, 7, or 14 days later, the mice were challenged with *S. pneumoniae* (10^3^ CFU). The number of bacteria was determined in lungs (A) and spleen (B) 30 h after *S. pneumoniae* challenge. The solid lines correspond to the median values. Results from a pool of two experiments are shown. ns, not significant. *, *P* < 0.05; ***, *P* < 0.001 (one-way ANOVA Kruskal-Wallis test).

To study the effects of exogenous iNKT cell activation on secondary bacterial infection, we established a mouse model of postinfluenza, highly invasive pneumococcal pneumonia. To this end, mice were intranasally (i.n.) infected with a sublethal dose (for the weight loss curve, see Fig. S1A in the supplemental material) of IAV/Scotland/20/74 (H3N2). At different time points thereafter, animals were challenged with a very low dose of the invasive *S. pneumoniae* serotype 1. While the dose of inoculated pneumococci was self-limiting as an infection in the absence of IAV, IAV-experienced mice were susceptible to pneumococcal challenge—as reflected by the presence of bacteria in the lungs and spleen (indicative of systemic dissemination) (Fig. 1). When mice were challenged at 4 days postinfluenza (dpi), bacteria were detected in lungs—albeit to a lesser extent than at 7 dpi (Fig. 1A). It is noteworthy that mice challenged at 4 dpi were resistant to invasive pneumococcal infection; this finding is in line with maintenance of lung barrier functions at that time point (Fig. 1B and Fig. S1B). A time course analysis indicated that susceptibility to pneumococcal infection peaked at 7 dpi, corresponding (along with 4 dpi) to the peak in the viral load (Fig. 1A and Fig. S1C). Mice remained susceptible to pneumococcal infection at 14 dpi.

### Early treatment with α-GalCer is associated with lower bacterial outgrowth in superinfected animals.

**FIG 2:**
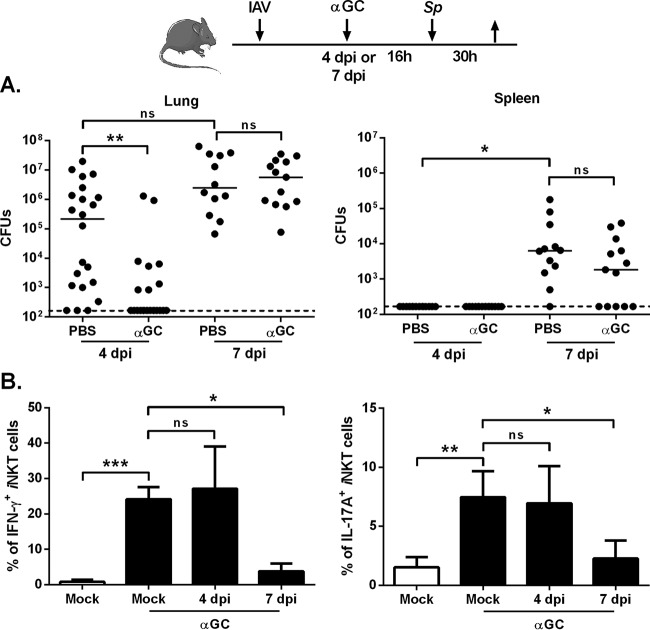
Effect of α-GalCer treatment during early influenza on secondary bacterial infection. (A) Overview of the procedure. Age-matched male mice were i.n. infected with IAV (30 PFU). Four or 7 days later, the mice were challenged with *S. pneumoniae* (*Sp* [10^3^ CFU]). Mice were i.n. treated with PBS or α-GalCer (2 µg/mouse) 16 h before the pneumococcal challenge. The number of CFU was determined in lungs and spleen 30 h after *S. pneumoniae* infection. The solid lines correspond to the median values. Results from a pool of three experiments are shown. (B) Mock-infected mice or IAV-infected mice (4 or 7 dpi) were i.n. treated with α-GalCer. Gated iNKT cells (TCR-β^+^ PBS57-loaded CD1d tetramer^+^) were analyzed for intracellular IFN-γ and IL-17Aproduction (16 h after α-GalCer inoculation). The *y* axis refers to the mean percentages of pulmonary iNKT cells positive for IFN-γ and IL-17A ± SD (*n* = 6 to 12, two pooled experiments). Of note, the absolute numbers of iNKT cells in the lungs of mock-infected and IAV-infected mice were similar (data not shown). ns, not significant. *, *P* < 0.05; **, *P* < 0.01; ***, *P* < 0.001 (one-way ANOVA Kruskal-Wallis test in panel B).

We next looked at whether α-GalCer could limit bacterial outgrowth and systemic dissemination in superinfected animals at the peak in viral load. In mice infected 4 days earlier with IAV, i.n. inoculation of α-GalCer 16 h before the secondary bacterial challenge was associated with a marked reduction in the pneumococcal load in the lungs (Fig. 2A). In stark contrast, administration of α-GalCer at 7 dpi failed to lower the number of pneumococci in the lungs and spleen, although for the latter, a nonsignificant reduction was observed. Similar data (reduced bacteria at 4 dpi but not 7 dpi) were obtained in a model of high-pathogenicity H1N1-pneumococcal infection (see Fig. S2A in the supplemental material). We then looked at whether α-GalCer’s effects at 4 and 7 dpi were related to differences in iNKT cell activation. Inoculation of α-GalCer at 4 dpi resulted in production of IFN-γ and IL-17A (cytokines known to have antipneumococcal activities) by pulmonary iNKT cells (32–34) (Fig. 2B; see Fig. S2B for the gating strategy). In stark contrast, administration of α-GalCer at 7 dpi failed to trigger cytokine production by iNKT cells. This finding was confirmed in the H1N1 IAV-pneumococcal infection system, although the inhibitory effect was less intense for IFN-γ (Fig. S2C). Taken as a whole, our results show that α-GalCer activates iNKT cells and triggers antipneumococcal immune defenses in superinfected animals but only when inoculated soon after the influenza infection.

### The lack of iNKT cell activation at 7 dpi is associated with a strongly reduced number of respiratory CD103^+^ cDCs.

**FIG 3:**
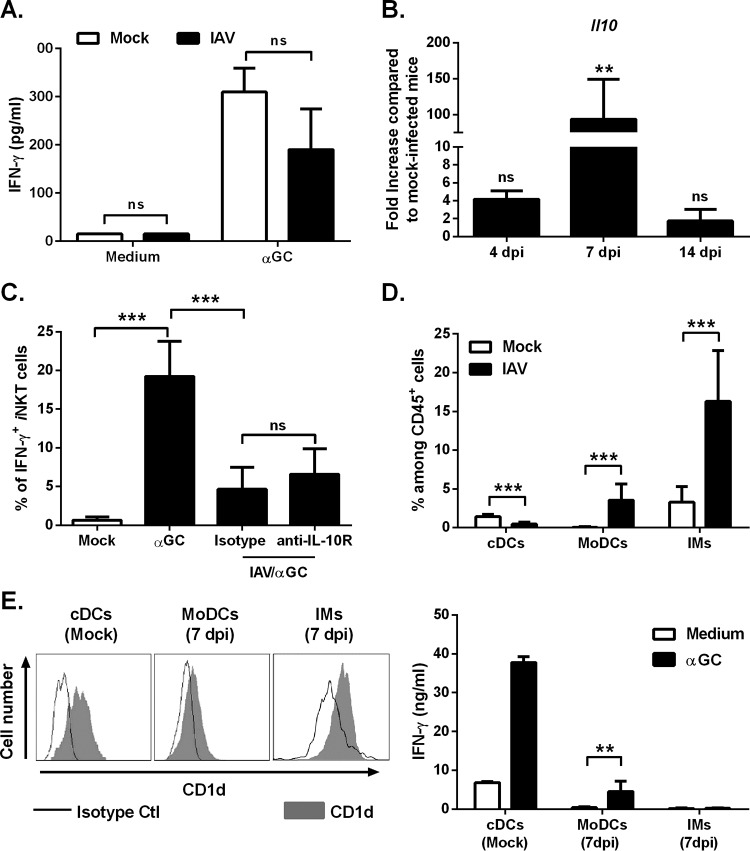
Role of the lung microenvironment on the inhibition of iNKT cell activation in the context of bacterial superinfection. (A) Pulmonary iNKT cells from mock-infected or IAV-infected mice were stimulated in an APC-free system with plate-coated CD1d/α-GalCer. The production of IFN-γ was quantified 48 h later. Results from a pool of two experiments are shown. (B) RNAs were extracted from the lungs of mock-infected and IAV-infected mice. IL-10 mRNA copy numbers were measured by quantitative RT-PCR. Data are expressed as fold increase ± SD over average gene expression in mock-treated mice (*n* = 5). (C) IAV-infected mice (6 dpi) were injected with the anti-IL-10 receptor (IL-10R) or the isotype control MAb (1 mg) 24 h before i.n. inoculation of α-GalCer. For each group, the percentages ± SD of IFN-γ- and IL-17A-producing iNKT cells (16 h after α-GalCer) are shown (*n* = 6 to 8, two pooled experiments). (D) The proportions ± SD of conventional DCs (cDCs), monocyte-derived DCs (MoDCs), and inflammatory monocytes (IMs) among CD45^+^ cells were determined in mock-infected and IAV-infected (7 dpi) mice (*n* = 8 to 10, three pooled experiments). (E, left panel) A representative histogram depicts the expression of CD1d on conventional DCs (naive mice), monocyte-derived DCs (7 dpi), and inflammatory monocytes (7 dpi). White peak, isotype control; gray peak, CD1d. (Right panel) Conventional DCs, monocyte-derived DCs, and inflammatory monocytes were sorted from mock-infected (cDCs) or IAV-infected (7 dpi) mice, incubated with α-GalCer for 1 h, and cocultured with naive iNKT cells. The concentration of IFN-γ present in the supernatant was determined after 48 h. Shown are the mean concentrations ± SD from two (cDCs) or three (MoDCs and IMs) experiments. ns, not significant. **, *P* < 0.01; ***, *P* < 0.001 (panels B and C, one-way ANOVA Kruskal-Wallis test).

In order to establish whether or not the lack of iNKT cell activation at 7 dpi was cell intrinsic, pulmonary iNKT cells from IAV-infected mice were exposed *ex vivo* to plate-bound α-GalCer-loaded CD1d. Influenza-experienced iNKT cells produced IFN-γ and IL-17A—indicating that they had retained their innate-like properties (Fig. 3A [data not shown]). This finding suggested that pulmonary factors have a role in the lack of *in vivo* cytokine production by iNKT cells in response to α-GalCer. One candidate factor is the immunosuppressive cytokine IL-10, which is known to be produced massively in the context of influenza infection (35, 36) (Fig. 3B). However, neutralizing IL-10 activities just before α-GalCer inoculation failed to rescue IFN-γ and IL-17A production by iNKT cells (Fig. 3C [data not shown]).

We have previously suggested that airway conventional DCs (cDCs [especially the CD103^+^ subset]) have a key role in the activation of pulmonary iNKT cells in response to the i.n. delivery of α-GalCer (33). In agreement with this, pulmonary iNKT cells from mice deficient for the basic leucine zipper transcription factor 3 (*Baft3*^−/−^), which lack CD103^+^ conventional DCs (37, 38 [data not shown]) produced less IFN-γ than wild-type controls (see Fig. S3A in the supplemental material). At 7 dpi, the proportion and absolute count of conventional DCs (including CD103^+^ cells) were much lower than in mock-infected mice (Fig. 3D and Fig. S3B and S3C). This phenomenon was not due to a local cell death since the frequency of dying/dead conventional DCs was not increased in the lungs during the course of IAV infection (Fig. S3D). In parallel, a strong influx of monocyte-derived DCs and inflammatory monocytes was observed in the lungs of IAV-infected mice (Fig. 3D). These cell types expressed CD1d on their surface, although the level of expression on monocyte-derived DCs was low (Fig. 3E, left panel). When exposed to α-GalCer *ex vivo*, monocyte-derived DCs and inflammatory monocytes induced less IFN-γ and IL-17A release by iNKT cells, relative to α-GalCer-exposed conventional DCs isolated from naive animals (Fig. 3E, right panel [data not shown]). Taken as a whole, our results show that the reduced number of conventional (CD103^+^) DCs from the lungs of IAV-infected mice (7 dpi) is associated with a lack of iNKT cell activation upon *in vivo* stimulation with α-GalCer.

### α-GalCer treatment during the resolution phase of influenza impairs pneumococcal outgrowth and dissemination in superinfected animals.

**FIG 4:**
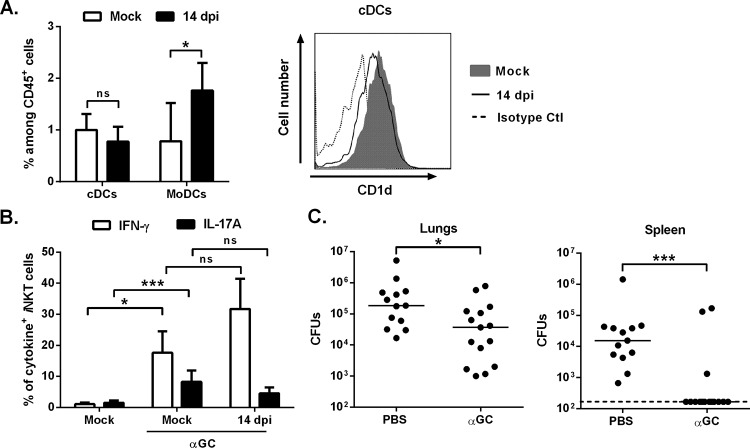
Effect of α-GalCer treatment during the resolution phase of influenza on secondary bacterial infection**.** (A, left panel) The frequencies (±SD) of pulmonary conventional DCs (cDCs) and monocyte-derived DCs (MoDCs) among CD45^+^ cells were determined in mock-infected and IAV-infected (14 dpi) mice. (Right panel) CD1d expression on conventional DCs is represented. Ctl, control. (B) Analysis of the frequency of pulmonary iNKT cells expressing IFN-γ and IL-17A from IAV-infected mice (14 dpi) in response to i.n. α-GalCer treatment (*n* = 10 to 12, three pooled experiments, mean ± SD). (C) IAV-infected mice were treated with α-GalCer (2 µg/mouse i.n.) 16 h before the *S. pneumoniae* challenge (14 dpi). The numbers of CFU in lungs and spleen were determined 30 h later. The solid lines correspond to the median values. Shown are cumulative data from two independent studies. ns, not significant. *, *P* < 0.05; ***, *P* < 0.001 (one-way ANOVA Kruskal-Wallis test for panel B).

We reasoned that the replenishment of respiratory conventional DCs in the lungs during the resolution phase of influenza might lead to enhanced resistance against superinfection upon α-GalCer treatment. The frequency/number of conventional DCs (both CD103^+^ and CD11b^+^) returned to basal levels at 14 dpi, while the proportion of monocyte-derived DCs and inflammatory monocytes fell (Fig. 4A, left panel, see Fig. S4A in the supplemental material [data not shown]).

The level of CD1d expression by respiratory conventional DCs at 14 dpi was similar to that observed in naive animals (Fig. 4A, right panel). In line with these findings, inoculation of α-GalCer at 14 dpi induced strong production of IFN-γ and IL-17A by iNKT cells (Fig. 4B and Fig. S4B, H1N1 IAV). Thus, conventional DCs accumulated in the lungs during the resolution phase of influenza—matching the window during which iNKT cells can be activated by α-GalCer.

We next wondered whether iNKT cell activation by α-GalCer is associated with enhanced antibacterial defenses. Inoculation of α-GalCer at 14 dpi lowered the pneumococcal count in the lungs (Fig. 4C and Fig. S4C, H1N1 IAV). More strikingly, α-GalCer almost fully abrogated the systemic dissemination of pneumococci. Similar data were obtained when mice were treated at 21 dpi (a time point at which mice are still susceptible to superinfection) (Fig. S4D).

### Combination treatment with dexamethasone and α-GalCer is associated with less intense pneumonia and a lower morbidity rate in IAV/pneumococcus-infected mice.

**FIG 5:**
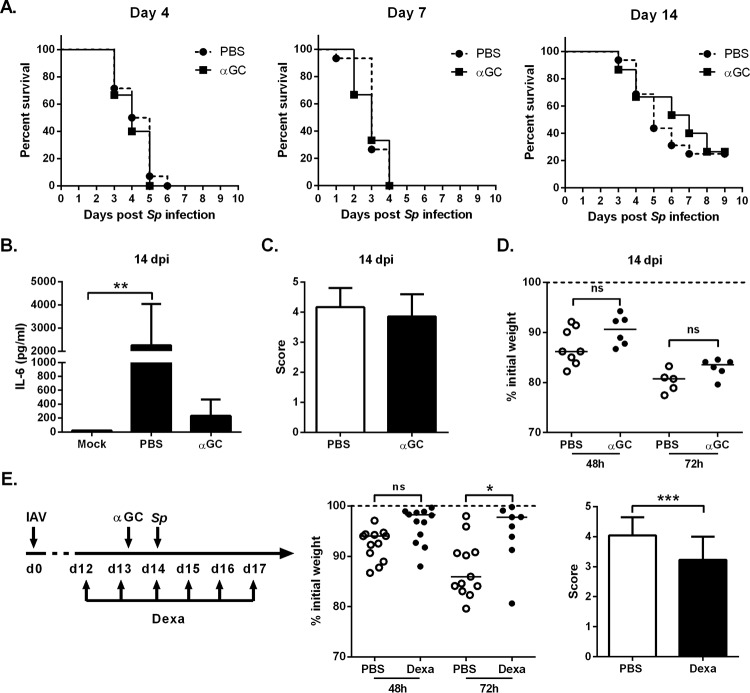
Effect of α-GalCer and dexamethasone treatment on the survival rate of superinfected animals**.** (A) IAV-infected mice were i.n. treated at different time points with PBS or α-GalCer (2 µg/mouse) (16 h before *S. pneumoniae* [*Sp*] challenge), and the survival of superinfected animals was monitored (*n* = 14 to 16/group, two pooled experiments). (B) IL-6 concentration in the serum of superinfected mice was quantified 24 h after *S. pneumoniae* infection. (C) For histopathologic examination, superinfected mice were killed 30 h after *S. pneumoniae* infection. Lung sections were scored blind for pneumonia with scores ranging from 0 to 5. (D) The body weights of the IAV-infected mice were measured 48 and 72 h after *S. pneumoniae* challenge and expressed relative to the weight at the time of bacterial challenge. (E) Overview of the procedure. IAV-infected mice were i.p. injected with dexamethasone (2.5 mg/kg) or vehicle 1 day before α-GalCer treatment (until day 17). Mice were challenged with *S. pneumoniae* at 14 dpi. (Middle panel) Modulations of body weights (relative to the weight before the bacterial challenge) are represented. (Right panel) Histopathological scores are indicated. (B, C, and E, right panel) Mean ± SD, *n* = 4 to 6 mice/group. One representative experiment out of two is shown. ns, not significant. *, *P* < 0.05; **, *P* < 0.01; *** *P* < 0.001 (one-way ANOVA Kruskal-Wallis test for panel B or Wilcoxon signed-rank test for panel E [right panel]).

Exacerbated lung injury, loss of respiratory function, and bacteremia are the main causes of mortality during postinfluenza superinfection (9, 39–42). We assessed the effect of α-GalCer treatment on the mortality rate in superinfected animals. Whatever the time point postinfluenza, α-GalCer treatment had no effect on the animals’ survival (Fig. 5A). This indicates that in our system, the size of the pneumococcal load is not correlated with the survival rate. Since the mortality rate in superinfected animals was lower at 14 dpi, we attempted to characterize the mechanisms leading to death at this time point. In line with the lower extent of bacterial dissemination in α-GalCer-treated superinfected mice (Fig. 4C), systemic inflammation (as measured by the serum concentration of IL-6) was lower in this group than in vehicle-treated counterparts (Fig. 5B). We therefore reasoned that excessive pneumonia (rather than septicemia) contributes strongly to mortality in α-GalCer-treated superinfected animals. A histological analysis of lung sections confirmed that severe pneumonia occurred in α-GalCer-treated superinfected animals to the same extent as in untreated superinfected animals—despite the lower bacterial load in the lungs (Fig. 5C). Similarly, α-GalCer did not improve the body weight loss after secondary pneumococcal infection (Fig. 5D). This prompted us to treat mice with the anti-inflammatory corticosteroid dexamethasone. It has been reported that combination treatment with dexamethasone and antibiotics lowers the extent of lung immune damage and mortality during secondary pneumococcal pneumonia (17, 42). To this end, mice were treated with dexamethasone 2 days before the *S. pneumoniae* challenge. We verified that this protocol does not alter iNKT cell activation in response to α-GalCer (see Fig. S5A in the supplemental material). The combined treatment was associated with a considerably smaller body weight loss during secondary bacterial infection and less intense pneumonia relative to mice treated with α-GalCer alone (Fig. 5E) or with dexamethasone alone (Fig. S5B [data not shown]). Despite this reduction in lung disease, combination treatment with α-GalCer and dexamethasone was not associated with a higher survival rate in superinfected animals (Fig. S5C). In summary, combined dexamethasone therapy and activation of iNKT cells is associated with lower bacterial outgrowth/dissemination, diminished pneumonia, and reduced morbidity (but no difference in survival) after IAV-pneumococcus infections.

## DISCUSSION

Severe influenza can lead to secondary bacterial infections with devastating consequences for human health. In view of the threat of a highly pathogenic influenza pandemic and increased antibiotic resistance, the development of alternative options for controlling bacterial superinfections (along with antivirals, antibiotics, and vaccines) is crucial. Very few studies have focused on the impact of local innate immunostimulators (such as Toll-like receptor [TLR] agonists) on postinfluenza secondary bacterial infections. This might be due to the fact that IAV induces the sustained desensitization of some innate sensors (e.g., TLRs) in lung tissue (43). However, our recent data indicated that the combination of local inoculation of flagellin (a TLR5 agonist) with antibiotic treatment can reduce the pulmonary pneumococcal burden after influenza (44). Furthermore, local treatment with MALP-2 (a TLR2/TLR6 agonist) lowers the load of pneumococci in the lungs and increases the survival rate in superinfected mice (45). In the present study, we investigated whether another strategy (namely, the exogenous activation of iNKT cells) can trigger local immunostimulation in the lung in the context of influenza.

The lipid α-GalCer is a potent iNKT cell agonist that is currently in clinical development (21–24). Data from our group and others indicate that α-GalCer provides prophylactic protection against lethal pneumococcal infections (an effect that depends on IFN-γ, IL-17A, and neutrophils) (31–33). We sought to establish whether this might also be the case in the context of prior influenza—even though the immunosuppressive environment imposed by IAV is known to contribute strongly to secondary bacterial infections (13–17) and to inhibition of iNKT cell activation in response to secondary pneumococcal challenge (36). Our results in H3N2 and H1N1 models showed that at 4 dpi (a time point when the viral load is high), treatment with α-GalCer is associated with a strong reduction of bacterial outgrowth in the lungs. The protective effect was associated with the activation of pulmonary iNKT cells (IFN-γ and IL-17A). This finding suggests that the effector cells (especially neutrophils) downstream of iNKT cells are functional at 4 dpi. In contrast, treatment with α-GalCer at 7 dpi (the peak in bacterial susceptibility) did not have an effect on secondary pneumococcal infection. Impairment of mucosal barrier function (which enables bacterial dissemination) and immune depression (as assessed by IL-10 synthesis, for example) also peak at 7 dpi. Functional changes in iNKT cells (including exhaustion) have been observed during cancer and infections (21, 46, 47). Under our experimental conditions, pulmonary iNKT cells from influenza-infected mice (7 dpi) became refractory *in vivo* to secondary stimulation with α-GalCer. An *ex vivo* analysis of iNKT cell functions showed that these cells retained their antigen-specific, innate-like properties in the context of IAV infection. Our data also rule out a dominant role for the immunosuppressive cytokine IL-10 in the inhibition of TCR-mediated iNKT cell activation. In fact, the strongly reduced number of conventional DCs (and especially CD103^+^ DCs) at 7 dpi might explain the lack of iNKT cell activation. Our data show that monocyte-derived DCs and inflammatory monocytes massively infiltrated the lungs during the acute phase of influenza but displayed low CD1d-based lipid-presenting capacities. Thus, at 7 dpi, IAV instigates mechanisms that affect lipid antigen-dependent activation of iNKT cells *in vivo*—thus hampering the potential therapeutic effect of α-GalCer. Among mechanisms leading to reduced frequency/number of pulmonary conventional DCs at 7 dpi, sustained emigration to the draining lymph nodes (48–50), rather than local cell death, is likely to be important.

We observed that IAV’s inhibitory effect was limited in time, since α-GalCer treatment at 14 dpi (during the viral recovery phase) and 21 dpi (data not shown) induced strong IFN-γ and IL-17A production by iNKT cells. This production was associated with repopulation of the lung tissue by CD1d-expressing (CD103^+^) conventional DCs. At 14 and 21 dpi, α-GalCer treatment in H3N2 and H1N1 models resulted in lower pneumococcal outgrowth in the lungs and reduced bacterial dissemination. The lack of a complete, effective antipneumococcal response following α-GalCer treatment in our system can be explained by the continuing depression of antibacterial functions in neutrophils and/or macrophages—i.e., cells known to act as downstream effectors of iNKT cells (21–24). The effect of α-GalCer at 14 dpi and thereafter was more prominent in terms of systemic bacterial translocation; this indicates a lower degree of immune depression in the periphery at these time points. The reduced local and systemic spread of pneumococci in α-GalCer-treated superinfected mice did not affect survival. Pneumonia, respiratory failure, and bacteremia are the major causes of death in IAV-superinfected patients (9, 39–42). Histological analysis of lung sections indicated the presence of severe postinfluenza bacterial pneumonia in vehicle-treated and α-GalCer-treated superinfected mice. This vigorous inflammatory response is usually associated with respiratory failure (9, 39–42) and is likely to have been the cause of death in our model of severe IAV-pneumococcal infection. Recent studies have shown that controlling bacterial outgrowth and exacerbated host inflammatory responses might be of particular value in reducing the negative outcomes of bacterial superinfections (17, 42). We took advantage of these findings and attempted to lower the morbidity and mortality rates of superinfected mice by treating mice with both α-GalCer and the anti-inflammatory corticoid dexamethasone. Combination treatment (at 14 dpi) resulted in a less intense IAV-pneumococcus-induced inflammatory response and a lower morbidity rate in the superinfected hosts. However, for reasons that have yet to be determined, this combination therapy did not have a significant effect on mouse survival. Our data therefore extend the results of animal studies (17, 42) in which strategies that lower the bacterial burden (e.g., antibiotics) do not impact mortality and thus must be combined with anti-inflammatory treatments that improve lung function. To the best of our knowledge, the present study is the first to show that the combination of an innate immunostimulator with an anti-inflammatory compound improves the outcome (bacterial outgrowth/dissemination, pneumonia, and morbidity) of postinfluenza bacterial superinfection. Targeting iNKT cells by combining specific agonists with other therapeutics (e.g., corticosteroids) might be of great value for the prevention and treatment of postinfluenza bacterial superinfections in humans. Consideration of the time window that effects are observed will be critical for future success in the human setting. Our study results also suggest that additional therapy is required to reduce mortality in superinfected hosts. The use of compounds that control lung injury and improve lung function (e.g., by reducing pulmonary edema) will be of value.

## MATERIALS AND METHODS

### Mice and ethics statement.

Eight-week-old male wild-type (WT) C57BL/6 mice were purchased from Janvier (Le Genest-St.-Isle, France). Mice were maintained in a biosafety level 2 facility in the Animal Resource Center at the Lille Pasteur Institute, All animal work conformed to the Lille Pasteur Institute animal care and use ethical guidelines (agreement no. AF 16/20090 and 00357.03). Mice lacking pulmonary CD103^+^ DCs (*Batf3*^−/−^) were described by Hildner and colleagues (37).

### Reagents and Abs.

α-GalCer was synthesized as described in reference 51, and dexamethasone was obtained from Sigma (St. Louis, MO). All labeled monoclonal antibodies (MAbs) were from BD Pharmingen (Le Pont de Claix, France). Neutralizing MAbs against IL-10 receptor (1B1.3A) and isotype controls were from BioXCell (West Lebanon, NH).

### Analysis of iNKT activation, purification, and culture.

Intracellular fluorescence-activated cell sorter (FACS) staining of iNKT cells was performed as previously described (50). Briefly, fixed cells were permeabilized and incubated with conjugated MAbs against IFN-γ or IL-17A or control rat IgG1 MAb. Cells were acquired and analyzed on an LSR Fortessa cytometer (Becton, Dickinson, Rungis, France) using the FACSDiva and FlowJo software. To purify iNKT cells, lung mononuclear cells were labeled with phycoerythrin (PE)-conjugated PBS57-loaded CD1d tetramer and fluorescein isothiocyanate (FITC)-conjugated anti-TCR-β Ab. Cells were sorted using a FACSAria (BD Biosciences). For culture experiments, pulmonary iNKT cells (5 × 10^3^ cells/well, purity of >98%) were stimulated with phorbol myristate acetate (PMA)/ionomycin or with plate-bound CD1d dimer loaded with α-GalCer.

### Infections and assessment of gene expression by quantitative RT-PCR.

Unless otherwise indicated, mice were intranasally (i.n. [50 µl]) infected with 30 PFU of the high-pathogenicity mouse-adapted H3N2 IAV strain Scotland/20/74 (50). In some cases, mice were infected with the high-pathogenicity H1N1 IAV strain WSN/33 (200 PFU). Assessment of gene expression by quantitative reverse transcription-PC (RT-PCR) was performed as described previously (50). Superinfection was as follows. Mice were infected (or not) with IAV, and 4, 7, 14, or 21 days later, animals were i.n. inoculated with 1 × 10^3^ CFU of *S. pneumoniae* serotype 1 (clinical isolate E1586) (33). To evaluate the effect of exogenous iNKT cell activation on superinfection, α-GalCer (2 µg/mouse) was i.n. inoculated 16 h before the secondary bacterial challenge. Enumeration of viable bacteria in lungs and spleen was determined 30 h after the *S. pneumoniae* challenge (33). Mouse survival and weight loss were measured daily.

### Analysis and purification of pulmonary conventional DCs, monocyte-derived DCs, and inflammatory monocytes.

To identify conventional DCs (cDCs) and monocyte-derived DCs, lung mononuclear cells were labeled with appropriate dilutions of FITC-conjugated anti-CD45, Brilliant Violet 421 anti-Siglec-F, allophycocyanin (APC)-H7-conjugated anti-Ly6G, phycoerythrin (PE)-Cy7-conjugated anti-CD11c, Alexa Fluor 700-conjugated anti-MHC class II, and APC-conjugated anti-CD64 Abs. Both cell types were identified as CD45^+^, Siglec-F^−^, CD11c^+^, and MHC class II^+^, cDCs being CD64^−^ and monocyte-derived DCs being CD64^+^ (52). Inflammatory monocytes were identified as CD45^+^, Siglec-F^−^, CD11b^+^, Ly6C^+^, and Ly6G^−^. These cells were negative for CD11c and expressed a small amount of MHC class II. Cells were sorted using the FACSAria (Becton, Dickinson). To analyze conventional dendritic cell death and apoptosis, FITC-conjugated annexin V Ab and propidium iodide were used.

### Coculture experiments.

Respiratory conventional DCs were sorted from naive mice, and monocyte-derived DCs and inflammatory monocytes were sorted from IAV-infected mice (7 dpi). These cells were exposed to α-GalCer (25 ng/ml) and then cocultured with cell-sorted naive pulmonary iNKT cells (2 × 10^4^ accessory cells/5 × 10^3^ iNKT cells/well). IFN-γ and IL-17A production was quantified by enzyme-linked immunosorbent assay (ELISA) 48 h later.

### Pulmonary histological analysis of superinfected mice and dexamethasome treatment.

Scoring of lung sections was performed by an experienced veterinary pathologist who was blind regarding the composition of the groups. Broncho-interstitial pneumonia was scored from 1 to 5 as described in references 36 and 50. To lower lung inflammation, IAV-infected mice were treated daily intraperitoneally (i.p.) with dexamethasone (2.5 mg/kg) from day 12 to day 15 (histological analysis) or from day 12 to day 17 (measurement of weight loss).

### Statistical analyses.

A Mann-Whitney Student’s unpaired *t* test or a Wilcoxon signed-rank test was used to compare two groups unless otherwise specified. Comparisons of more than two groups with each other were analyzed by one-way ANOVA Kruskal-Wallis test (nonparametric), followed by Dunn’s posttest (PRISM v6 software; GraphPad). Survival of mice was compared using Kaplan-Meier analysis and log-rank test. Results are expressed as the mean ± standard deviation (SD) unless otherwise stated. A *P* value of <0.05 was considered significant.

## SUPPLEMENTAL MATERIAL

Figure S1  (A) Age-matched mice were i.n. infected with IAV (30 PFU), and weight loss was measured every 2 days. (B) Kinetics of epithelial barrier damage during influenza as assessed by the concentrations of protein in the bronchoalveolar lavage fluids (mean ± SD, *n* = 5 to 7 mice/group). (C) RNAs were extracted from the lungs of mock-treated and IAV-infected mice. Differences in viral loads between animal groups were determined by quantitative RT-PCR. Data are expressed as cycle threshold (Ct) values. The dashed line represents the detection threshold. Data represent the mean ± SD (*n* = 5 mice/group). ns, not significant. **, *P* < 0.01 (one-way ANOVA Kruskal-Wallis test). One representative experiment out of two is shown. Download Figure S1, TIF file, 0.1 MB

Figure S2  (A) The same procedure as in Fig. 2, but mice were infected with IAV/WSN/33 (H1N1, 200 PFU). (B) The TCR-β^+^ PBS57-loaded CD1d tetramer^+^ cells were analyzed for intracellular IFN-γ and IL-17 production 16 h after α-GalCer inoculation (mock-infected versus IAV-infected mice, 4 dpi). (C) Analysis of cytokine production by iNKT cells was performed at 7 dpi. One representative experiment out of two is shown (*n* = 4 to 8). ns, not significant. *, *P* < 0.05; **, *P* < 0.01. Download Figure S2, TIF file, 0.1 MB

Figure S3  (A) α-GalCer was administered i.n. to wild-type or *Baft3*^−/−^ mice, and 16 h later, the frequency of IFN-γ-producing iNKT cells was determined (*n* = 7, mean ± SD). ***, *P* < 0.001 (Mann-Whitney Student’s unpaired *t* test). (B) Gating strategies for detection of conventional DCs (mock-infected mice versus IAV-infected mice, 7 dpi). The CD45^+^ Siglec F^−^ CD11c^+^ MHC class II^+^ population was gated, and cells in this gate were identified as conventional DCs (CD64^−^). Conventional DCs were discriminated on the basis of CD103 and CD11b expression. Representative flow cytometry plots are shown. (C) The number of pulmonary CD103^+^ conventional DCs is indicated in mock-infected and IAV-infected mice (IAV/H3N2/Scotland/20/74 and IAV/H1N1/WSN/33). One representative experiment out of two is shown (*n* = 5, mean ± SD). *, *P* < 0.05; **, *P* < 0.01 (one-way ANOVA Kruskal-Wallis test). (D) The mortality rates of pulmonary conventional DCs were determined at 2, 4, and 6 dpi. Cells positive for propidium iodide were considered dead, and cells positive for annexin V were considered apoptotic. Representative plots are shown. Download Figure S3, TIF file, 0.1 MB

Figure S4  (A) Gating strategies for detection of conventional CD103^+^ and CD11b^+^ DCs (14 dpi). (B) The same procedure as in Fig. 4B, but mice were infected with IAV/WSN/33 (H1N1). Shown are the frequencies of pulmonary iNKT cells expressing IFN-γ and IL-17A. A representative experiment out of three (*n* = 4, mean ± SD) is shown. ns, not significant. *, *P* < 0.05; **, *P* < 0.01 (one-way ANOVA Kruskal-Wallis test). (C and D) IAV (H1N1 or H3N2)-infected mice were treated at 14 dpi (C) or at 21 dpi (D) with α-GalCer (2 µg/mouse i.n.) 16 h before *S. pneumoniae* challenge. The number of bacteria in lungs and spleen was determined 30 h later. The solid lines correspond to the median values. Shown is a representative experiment out of two. *, *P* < 0.05; **, *P* < 0.01. Download Figure S4, TIF file, 0.1 MB

Figure S5  (A) IAV-infected mice were i.p. injected with dexamethasone (2.5 mg/kg) or vehicle 1 day before α-GalCer treatment and at the time of α-GalCer treatment. Production of IFN-γ and IL-17A was analyzed 16 h later. One experiment out of two is shown (*n* = 4, mean ± SD). (B, left panel) Modulations of body weights (relative to the weight before the bacterial challenge) are represented (PBS-treated mice versus dexamethasone-treated mice, without α-GalCer). (Right panel) Effect of α-GalCer, dexamethasone, or α-GalCer plus dexamethasone treatment on the survival rate of superinfected animals (*n* = 8/group). One experiment out of two is shown. ns, not significant. Download Figure S5, TIF file, 0.1 MB
